# Evidence of Significant Intratumoral Transcriptomic Heterogeneity in Non-functioning Pituitary Adenomas Based on Location and Texture

**DOI:** 10.7759/cureus.75649

**Published:** 2024-12-13

**Authors:** Faraz Behzadi, Parker L Woldt, John T Tsiang, Brandon M Zsigray, Diego D Luy, Meharvan Singh, Peter Larsen, Anand V Germanwala

**Affiliations:** 1 Neurological Surgery, Loyola University Medical Center, Maywood, USA; 2 Cellular and Molecular Physiology, Loyola University Medical Center, Maywood, USA; 3 Genetics, Loyola University Medical Center, Maywood, USA

**Keywords:** differential gene expression, pituitary adenomas, transcriptomics, tumor cell biology, tumor texture

## Abstract

Introduction

Surgical resection remains a standard treatment of non-functioning pituitary adenomas (NFPA). These tumors have significant intratumoral variability of growth rates and texture hardness. This preliminary study aims to identify variations in gene expression of different locations and textures within the same tumor to better explain tumor pathophysiology.

Methods

NFPA tissue samples were collected from four non-consecutive surgical adult patients undergoing endoscopic endonasal resection and were sent for next-generation transcriptomics. Significantly differentially expressed (SDE) genes were analyzed and categorized using ontology within different locations of the tumor, tumor hardness, and across patients.

Results

Around 164 SDE genes were identified: 264 across tumor hardness and 68 across location marginality (core vs. edge). A total of 132 gene ontology annotations were matched to all SDE genes. Most of these annotations involved a combination of cell metabolism, cell-cell interactions, and cell division.

Conclusions

There was significant evidence of variations and uniqueness in intratumor genetic heterogeneity within different locations, tumor textures, and across patients. The tumor edge expressed higher cell-cell interaction genes such as cadherin-binding proteins. Soft portions of the tumor experienced an upregulation of anaerobic metabolism and cell division genes. The uniqueness of gene expressions can be tested for biological function, prospectively, with the potential targets for gene therapy.

## Introduction

Tumors of the central nervous system (CNS) have shown evidence of intratumoral genetic heterogeneity, a characteristic that can be highly informative regarding the incidence of tumor recurrence and the development of more effective non-surgical treatments [1-3]. One of the more common benign tumors of the CNS is non-functioning pituitary adenomas (NFPA), which comprise 10-15% of all intracranial tumors [4-6]. Given the notable phenotypic intratumoral differences observed through magnetic resonance imaging (MRI) and intraoperative findings, some studies have implied differences in microscopic pathology and gene expression profiles between distinct intratumoral locations of NFPA cells (e.g., tumor edge vs. core) and distinct intratumoral textures [7]. The genomic profiling of NFPAs can not only provide insight for the development of less invasive targeted gene-therapy treatment modalities but also may potentially predict tumor recurrence and invasiveness. These findings can significantly improve postoperative expectations and overall patient care [2,3].

Efforts of prior whole-exome sequencing studies of NFPAs have found low intertumoral mutation rates, consistent with the relatively slow-growing and benign nature of these tumors [8]. However, more subtle findings, such as the clinically silent production of luteinizing hormone (LH) and follicular stimulating hormone (FSH) sometimes seen with NFPAs, are only highly correlated to the differences seen in gene expression analysis [9]. Intratumoral heterogeneity in NFPAs, therefore, may be most emphasized and best explainable at the gene expression/transcriptomics level.

Original differential gene expression studies of pituitary adenomas have used microarrays and mostly focused on functional adenomas (e.g., prolactinomas), of which gene expressions are more readily differentiable [10,11]. Recent next-generation sequencing and transcriptomic studies have also been able to stratify differential gene expression patterns seen between functioning adenomas and NFPAs into meaningful gene clusters that correlated strongly with the type of secretory hormones [12]. Some have focused primarily on the differential gene expression of gonadotrophs, which has identified a unique gene regulatory network for NFPAs (centered around the pituitary adenoma stem cell (PASC) gene) and a potentially therapeutically targetable gene, ANXA-2 [13]. There is, however, a lack of gene expression studies on intratumoral genetic heterogeneity based on core and edge locations solely amongst the NFPA tumor type and between areas with different textures, adhesiveness, and invasiveness, of which differences may predict surgical outcomes and tumor recurrence [14].

This study aims to evaluate genetic heterogeneity in non-functioning pituitary adenomas (NFPAs) by analyzing differential gene expression across tumor locations (core vs. edge) and textures (firm vs. soft) to identify biomarkers and potential therapeutic targets. In the clinical setting, adenomas have heterogeneity between patients (for example, some adenomas invade the cavernous sinus and diaphragma while others respect those structures) and within the tumor itself. Intratumoral heterogeneity may explain growth patterns of adenomas, perhaps with a leading edge at the margin for tumor growth and invasion compared to the core. This may explain growth patterns and provide a better understanding for adenoma pathophysiology and refute the notion that adenomas are a monomorphic cell population. We hope our findings here will lead to future investigations on the existence of meaningful biologic gene correlates and patterns of expression as a basis to define models of predicting tumor recurrence and to discover targeted gene therapies for more challenging invasive or adhesive NFPAs.

## Materials and methods

This article was previously posted to the Research Square preprint server on March 5, 2024. This study design was performed in line with the principles of the Declaration of Helsinki and was conducted at the Loyola University Medical Center, Maywood, United States. Approval was granted by the Institutional Review Board (IRB# LU 215922). Informed consent was obtained from all individual participants included in the study. Four consecutive patients requiring surgical endoscopic resection of non-functioning pituitary adenomas were selected. This study was conducted from April 22, 2022, to April 22, 2023.

NFPA tissue samples were collected from all four adult (≥ 18 years of age) patients presenting for endoscopic endonasal transsphenoidal surgical resection. All patients were symptomatic, but none had clinical evidence of hormonal dysregulation on preoperative serum endocrine panels. Once the diagnosis of pituitary adenoma was confirmed intraoperatively by pathology, two samples were collected intraoperatively: one from the edge of the tumor (the immediate 1 cm superficial anterior sellar surface of the tumor upon encounter during surgery) and one from the core (the best intraoperative estimation of the center of the tumor using stereotactic navigation). A total of eight samples from four patients were, therefore, included in this study. Samples were placed on ice in the operating room and transported within 10 minutes on average to the laboratory where they were immediately frozen and stored. The diagnosis of NFPA was made on final pathology with corresponding negative hormonal immunohistochemical staining. 

Tumors were transported to the laboratory for cell homogenization after being collected from storage. No more than 30 mg of tissue per sample was placed in a mortar, soaked with liquid nitrogen, and ground with a pestle. Using 500 μL of phosphate-buffered saline (PBS), the samples were further broken down mechanically with repeated pipetting. Using an additional 500 μL of PBS, the samples were collected from the mortar, centrifuged at 10,000 g-forces for one minute in a microcentrifuge tube, the extra liquid was disposed of, and the sample was placed in a -80°C freezer for temporary storage. 

DNase digestion and RNA isolation were performed for each sample using a QIAGEN RNeasy Mini Kit (Cat. No./ID: 74104) including all optional steps. RNA yield was recorded using a Thermo Scientific NanoDrop One C Microvolume UV-Vis Spectrophotometer (Thermo Fisher Scientific INC., Waltham, United States). After recording the volume, concentration, and A260/280 and A260/230 ratios for each sample, samples were subsequently given de-identified labels per patient and tumor location and transported on dry ice to the Loyola Genomics Facility for transcriptomic analysis. 

Besides the aforementioned tumor sampling marginality location: core versus edge, tumor hardness was defined as soft if only a two-suction technique was used for resection (grades 1 and 2 based on Rutkowski et al.) and firm if curettes and/or an ultrasonic aspirator were used for resection of the tumor (grades 3-5) [15]. The three main variables compared in groups against gene expression profiles are sampling marginality, tumor hardness, and individual patients. Other variables reviewed but not compared include age, sex, race, presenting symptoms, time from adenoma diagnosis to surgical treatment, tumor size, and gross total resection rates.

RNAseq library generation and DNA sequencing

Digital gene expression libraries were generated from total RNA using the “QIAseq UPX 3’ Transcriptome Kit” (catalog number 33088) following standard manufacturer protocols (QIAGEN QIaseq UPX 3’ Transcriptome Handbook, 01/2021, QIAGEN, Germantown, United States), and QC of the library was performed using the Agilent Bioanalyzer ‘High Sensitivity dsDNA’ cartridge (Agilent Technologies, Santa Clara, United States). Libraries were sequenced on an ‘Illumina MiSeq’ instrument (Illumina, San Diego, United States) using a ‘V3 600bp’ flow cell (Catalog number MS-102-3003).

RNAseq data analysis

Gene expression was determined from DNAseq data using ‘CLC Genomics Workbench v22’ (QIAGEN, Germantown, United States) with the ‘Quantify QIAseq UPX 3 v0.58’’ template workflow, using QIAseq UPX hg3 geneses and manufacturer-recommended parameter settings. Gene expression data was log2 transformed and normalized by quantiles. Significantly differentially expressed (SDE) genes to resolve tumor location across patients were identified using 2-factor ANOVA considering factors for ‘Patient’ and ‘Location’ (10,000 iterations and Benjamini-Hochberg (BH)-adjusted p-values < 0.05). SDE genes between ‘Soft’ and ‘Firm’ phenotypes were calculated by T-test (10K permutations, BH-adjusted p-values < 0.05). Computer program ‘R’ (R Foundation for Statistical Computing, Vienna, Austria, https://www.R-project.org/) and the package ‘Limma’ were used to draw statistical conclusions .

Enriched GO categories, relative to the distribution of gene function annotation in the annotated human genome, for SDE gene sets were determined using the “GO Enrichment Analysis” tool (https://geneontology.org/) for ‘PANTHER GO-slim’ ontologies (PANTHERv18.0,’ ‘PANTHER Overrepresentation Test’ with Fisher’s exact test, uncorrected p-values < 0.001). Identified GO-Slim annotations were further grouped into broad categories for comparing results across treatments: cell division, cell-cell interaction, cytoskeletal, localization, metabolism, organelle, protein biosynthesis, regulation, signaling, and transport.

## Results

**Figure 1:**
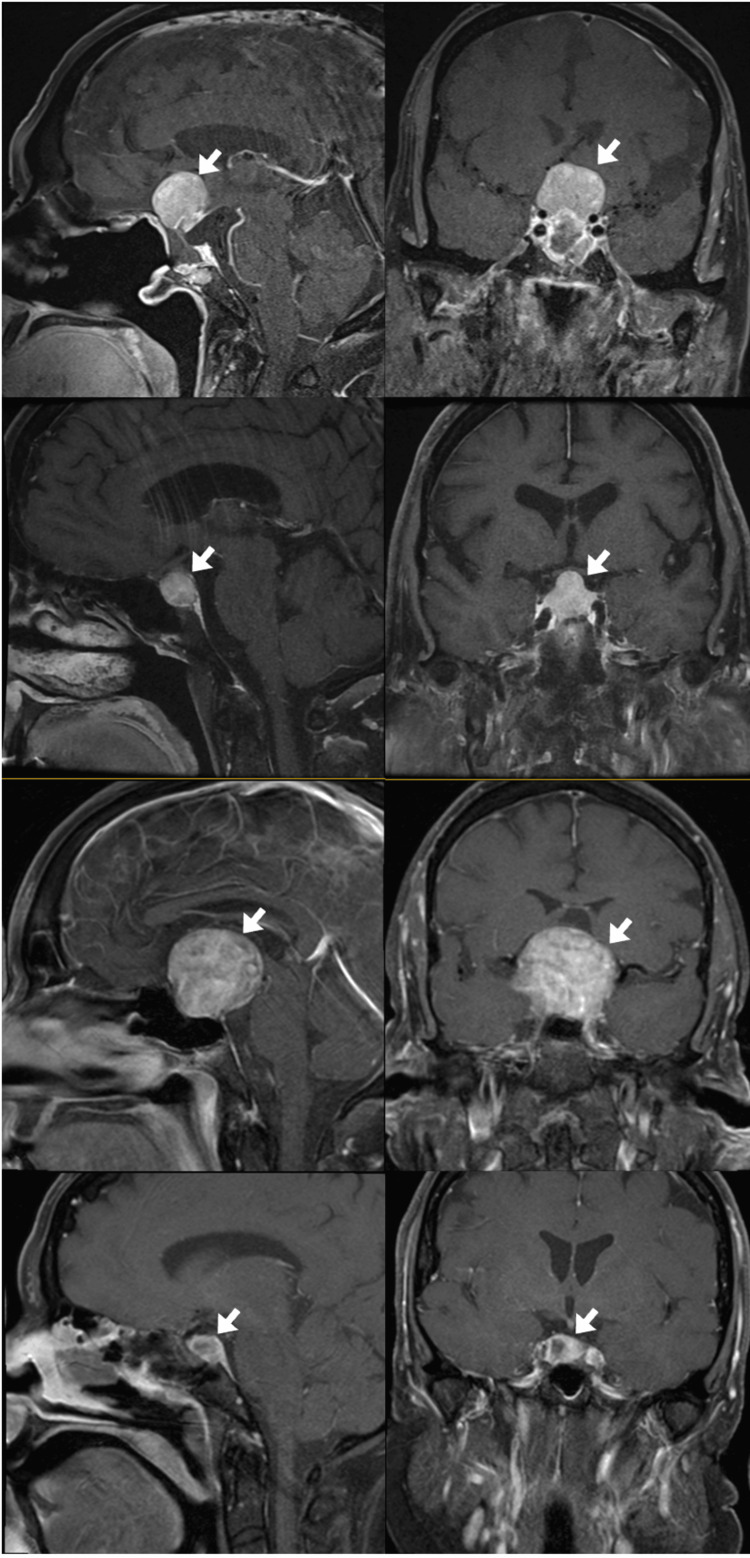
Preoperative dedicated-pituitary MRIs of the pituitary adenoma Sagittal (A,C,E,G) and coronal (B,D,F,H) MRIs for each patient (#1: A/B; #2: C/D; #3: E/F; #4: G/H) representing heterogenous intratumoral intensity across patients.

A total of four adult patients and eight pituitary adenoma tissue samples were included in this study. Clinical demographics revealed an average age of 67 years (standard deviation of 11 years), three (75%) of whom were males, three (75%) of whom had symptomatic presentations, including three (75%) with visual field deficits from chiasmatic compression, and one (25%) with a sixth nerve palsy and memory changes. None of these patients experienced pituitary apoplexy. None of the patients had prior resections. The average tumor volume of these patients was 21.16 cm³. We have included sagittal and coronal dedicated pituitary MRIs of our patients preoperatively to show the heterogeneity of tumors on imaging (Figure 1). A demographics table is provided (Table 1). Signal intensity within the tumor has significant variation. Two (50%) of these patients underwent gross total resection without any long-term complications. Two (50%) patients experienced an intraoperative cerebrospinal fluid leak that was able to be repaired. Two (50%) of the patients experienced postoperative hormonal deficits requiring hormonal replacement therapy with steroids. All (100%) patients had improvement in their preoperative visual disturbances and/or neurologic deficit at six weeks follow-up.

**Table 1:** Patient demographics

Total Patients N = 4	
		Age, years	
Mean (SD)	67 (11)
Sex, n(%)	
Female	1 (25)
Race n(%)	
White	3 (75)
Black	0 (0)
Hispanic	1 (25)
Asian	0 (0)
Time diagnosis to surgery, days	
Mean (SD)	76 (112)
Size, cm^3^	
Mean (SD)	21.2 (15)
Symptoms, n(%)	
Headaches	0 (0)
Visual deficits	3 (75)
Other neurologic deficits	1 (25)
Tumor marginality, n(%)	Samples = 8
Core	4 (50)
Edge	4 (50)
Tumor hardness, n(%)	Samples = 8
Firm	4 (50)
Soft	4 (50)
Gross tumor resection, n(%)	
Yes	2 (50)

Differential gene expression as a function of location

**Figure 2:**
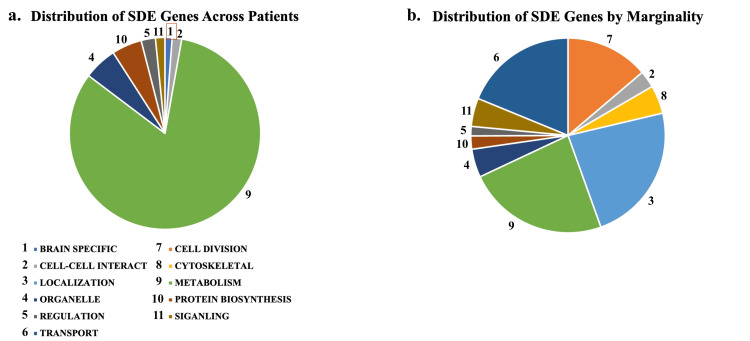
Pie chart findings a: pie chart distribution of SDE genes across patients; b: pie chart distribution of SDE genes by marginality SDE: significantly differentially expressed

**Figure 3:**
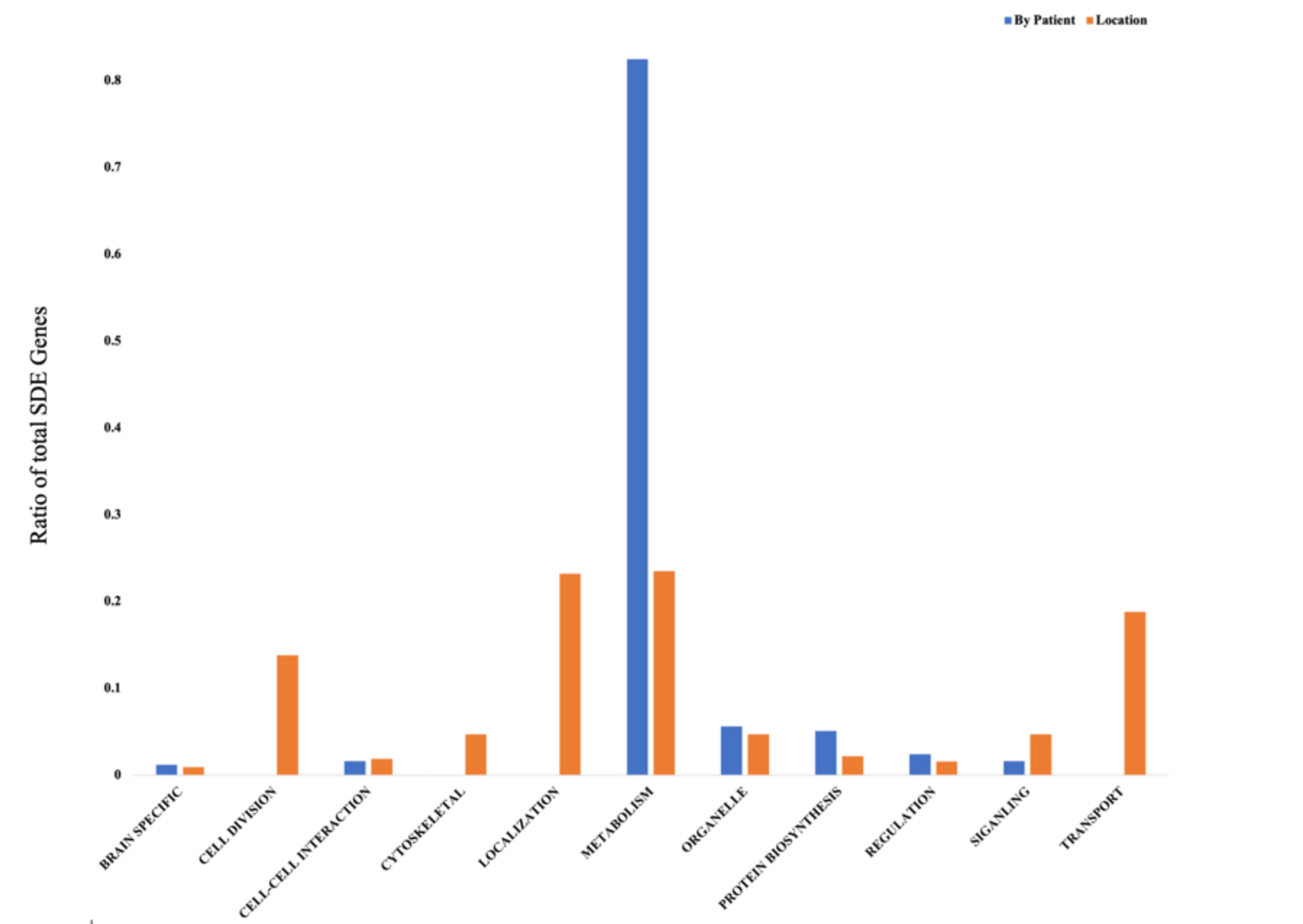
Bar plots of variations in ontologic categories of SDE genes across patients and by marginality SDE: significantly differentially expressed

Results of 2-Factor ANOVA (factors ‘Patient’ and ‘Location’): 164 SDEs as a function of ‘Patient,’ 68 SDEs as a function of ‘Location’ and 19 SDEs in common between factors. Around 47 PANTHER GO-Slim annotations were found to be enriched in genes SDE by patient. The primary difference between patients was in genes annotated as metabolic in nature (82% of all patients associated SDE genes, followed by organelle (5.6%)). Fifty-six GO-Slim annotations were found to be enriched in genes SDE by tumor location. Differences in gene expression between ‘core’ and ‘edge’ locations were also predominantly annotated as metabolic (23%), followed closely by genes annotated with localization functions (23%), then intracellular transport mechanisms (19%), and cell division (14%) (Figures 2, 3, Tables 2, 3).

**Table 2:** Gene ontology of differential gene expression of samples by marginality location GO: gene ontology

Category	Gene Ontology	Number of All Genes	Number of Differentially Expressed	P-Value
Signaling	cyclin-dependent protein kinase activity (GO:0097472)	70	2	2.03E-02
	intracellular signal transduction (GO:0035556)	820	6	4.19E-02
	calcium ion binding (GO:0005509)	168	3	1.56E-02
	regulation of intracellular signal transduction (GO:1902531)	415	4	4.04E-02
Protein biosynthesis	translation regulator activity (GO:0045182)	69	3	1.35E-03
	protein-containing complex assembly (GO:0065003)	367	4	2.75E-02
Cell-cell Interaction	cell adhesion molecule binding (GO:0050839)	141	3	9.81E-03
	cell-cell adhesion (GO:0098609)	214	3	2.93E-02
	cadherin binding (GO:0045296)	50	3	5.34E-04
Regulation	RNA binding (GO:0003723)	617	5	4.29E-02
Metabolism	positive regulation of cellular biosynthetic process (GO:0031328)	279	4	1.12E-02
	positive regulation of biosynthetic process (GO:0009891)	282	4	1.17E-02
	cellular biosynthetic process (GO:0044249)	2731	14	3.89E-02
	organic substance biosynthetic process (GO:1901576)	2773	14	4.34E-02
	biosynthetic process (GO:0009058)	2778	14	4.40E-02
	organophosphate biosynthetic process (GO:0090407)	234	3	3.66E-02
	phosphate-containing compound metabolic process (GO:0006796)	960	7	2.92E-02
	phosphorus metabolic process (GO:0006793)	965	7	2.99E-02
	ATP metabolic process (GO:0046034)	68	2	1.92E-02
	organonitrogen compound biosynthetic process (GO:1901566)	692	6	2.05E-02
Localization	localization (GO:0051179)	2469	15	7.73E-03
	establishment of localization in cell (GO:0051649)	943	9	2.49E-03
	cellular localization (GO:0051641)	1159	9	9.41E-03
	macromolecule localization (GO:0033036)	909	7	2.25E-02
	establishment of localization (GO:0051234)	1993	15	9.77E-04
	protein localization (GO:0008104)	748	6	2.86E-02
	establishment of protein localization (GO:0045184)	414	5	9.39E-03
	cellular component biogenesis (GO:0044085)	1024	8	1.38E-02
Organelle	establishment of organelle localization (GO:0051656)	239	3	3.86E-02
	organelle fission (GO:0048285)	343	4	2.22E-02
	cellular component assembly (GO:0022607)	896	8	6.47E-03
Cell division	mitotic sister chromatid segregation (GO:0000070)	56	3	7.41E-04
	sister chromatid segregation (GO:0000819)	70	3	1.40E-03
	nuclear chromosome segregation (GO:0098813)	101	3	3.93E-03
	chromosome segregation (GO:0007059)	118	3	6.04E-03
	chromosome organization (GO:0051276)	256	4	8.40E-03
	mitotic cell cycle (GO:0000278)	271	4	1.02E-02
	mitotic cell cycle process (GO:1903047)	271	4	1.02E-02
	mitotic nuclear division (GO:0140014)	271	4	1.02E-02
	cell cycle process (GO:0022402)	442	6	2.53E-03
	cell cycle (GO:0007049)	476	6	3.64E-03
	nuclear division (GO:0000280)	326	4	1.88E-02
Cytoskeletal	microtubule cytoskeleton organization (GO:0000226)	373	4	2.90E-02
	microtubule-based process (GO:0007017)	488	5	1.80E-02
	microtubule binding (GO:0008017)	142	3	1.00E-02
	tubulin binding (GO:0015631)	177	3	1.79E-02
Transport	nuclear transport (GO:0051169)	111	3	5.11E-03
	nucleocytoplasmic transport (GO:0006913)	111	3	5.11E-03
	Golgi vesicle transport (GO:0048193)	295	4	1.35E-02
	intracellular protein transport (GO:0006886)	323	4	1.83E-02
	protein transport (GO:0015031)	405	5	8.59E-03
	nitrogen compound transport (GO:0071705)	567	7	1.89E-03
	transport (GO:0006810)	1962	13	6.71E-03
	organic substance transport (GO:0071702)	736	7	7.80E-03
	intracellular transport (GO:0046907)	787	7	1.10E-02
	vesicle-mediated transport (GO:0016192)	815	7	1.32E-02

**Table 3:** Gene ontology of differentially expressed genes of samples across patients GO: gene ontology

Category	Gene Ontology	Number of All Genes	Number of Differentially Expressed	P-Value
Brain specific	amyloid-beta binding (GO:0001540)	11	3	7.77E-05
	neurotransmitter receptor activity (GO:0030594)	101	3	3.76E-02
	neurotransmitter binding (GO:0042165)	102	3	3.85E-02
Signaling	GTPase activity (GO:0003924)	368	7	1.82E-02
	TOR signaling (GO:0031929)	46	3	4.73E-03
	cell surface receptor signaling pathway (GO:0007166)	999	2	2.25E-02
Protein synthesis	structural constituent of ribosome (GO:0003735)	111	3	4.74E-02
	ribosomal small subunit biogenesis (GO:0042274)	52	3	6.62E-03
	ribosome biogenesis (GO:0042254)	167	4	3.39E-02
	protein metabolic process (GO:0019538)	2090	24	1.57E-02
	negative regulation of protein metabolic process (GO:0051248)	176	4	3.98E-02
Cell-cell interaction	multicellular organismal process (GO:0032501)	1439	5	4.72E-02
	cell junction assembly (GO:0034329)	90	3	2.82E-02
	cell junction organization (GO:0034330)	146	4	2.22E-02
Regulation	mRNA binding (GO:0003729)	182	4	4.41E-02
	regulation of cellular catabolic process (GO:0031329)	148	4	2.32E-02
	translational elongation (GO:0006414)	254	5	3.82E-02
	translation (GO:0006412)	254	5	3.82E-02
Metabolism	pyrophosphatase activity (GO:0016462)	696	12	4.92E-03
	hydrolase activity acting on anhydrides (GO:0016818)	699	12	5.09E-03
	hydrolase activity acting on acid anhydrides (GO:0016817)	699	12	5.09E-03
	nucleoside-triphosphatase activity (GO:0017111)	655	11	8.43E-03
	hydrolase activity (GO:0016787)	1739	19	4.68E-02
	glycoprotein biosynthetic process (GO:0009101)	113	3	4.95E-02
	regulation of catabolic process (GO:0009894)	163	4	3.14E-02
	amide biosynthetic process (GO:0043604)	295	6	2.10E-02
	regulation of hydrolase activity (GO:0051336)	298	6	2.20E-02
	peptide biosynthetic process (GO:0043043)	257	5	3.99E-02
	peptide metabolic process (GO:0006518)	318	6	2.89E-02
	organonitrogen compound biosynthetic process (GO:1901566)	692	12	4.71E-03
	cellular amide metabolic process (GO:0043603)	412	7	3.11E-02
	cellular protein metabolic process (GO:0044267)	1765	21	1.65E-02
	organonitrogen compound metabolic process (GO:1901564)	2573	29	1.01E-02
	cellular nitrogen compound metabolic process (GO:0034641)	3265	32	4.27E-02
	cellular macromolecule metabolic process (GO:0044260)	3864	37	4.01E-02
	nitrogen compound metabolic process (GO:0006807)	5018	48	1.85E-02
	primary metabolic process (GO:0044238)	5251	50	1.73E-02
	cellular metabolic process (GO:0044237)	5332	50	2.27E-02
	macromolecule metabolic process (GO:0043170)	4815	45	3.35E-02
	organic substance metabolic process (GO:0071704)	5685	52	3.09E-02
	metabolic process (GO:0008152)	5907	53	4.07E-02
	cellular process (GO:0009987)	10019	84	3.57E-02
Organelle	non-membrane-bounded organelle assembly (GO:0140694)	150	4	2.42E-02
	organelle assembly (GO:0070925)	348	6	4.18E-02
	cellular protein-containing complex assembly (GO:0034622)	348	6	4.18E-02
	cellular component biogenesis (GO:0044085)	1024	14	1.70E-02
	cellular component assembly (GO:0022607)	896	12	3.02E-02

Results for comparison between ‘firm’ and ‘soft’ phenotypes

**Figure 4:**
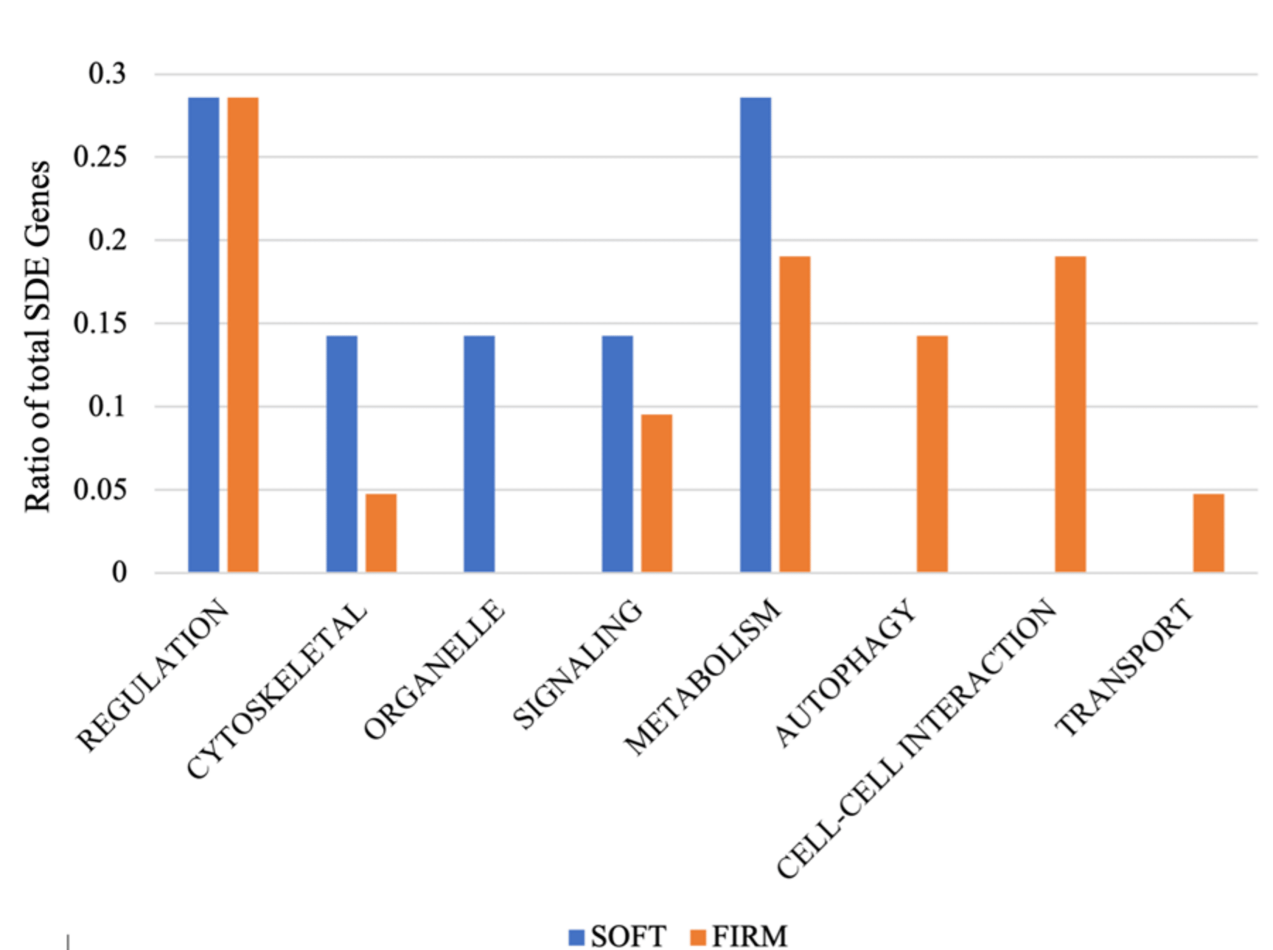
Bar plot of variations in ontologic categories of SDE genes by tumor hardness (soft vs. firm) SDE: significantly differentially expressed

Results of the T-test SDE 264 significantly differentially expressed genes between ‘firm’ and ‘soft’ tumor phenotypes were identified by T-test: 235 genes up-regulated in ‘firm’ and 128 genes up-regulated in ‘soft.’ Genes up-regulated in ‘soft’ tumor phenotypes were predominantly annotated as metabolic or regulatory in nature (29% each), while genes up-regulated in ‘firm’ tumor phenotypes were predominately regulatory (29%) and metabolic or cell-cell interactions (19% each). Around 29 PANTHER GO-Slim annotations were identified as significantly enriched in set of differentially expressed genes of which 21 annotations enriched from up-regulated in ‘firm’ and eight annotations enriched from up-regulated in ‘soft’ (Figure 4, Table 4).

**Table 4:** Gene ontology of differential gene expression of samples by tumor hardness GO: gene ontology

	Category	Gene Ontology	Number of All Genes	Number of Differentially Expressed
Up regulated in soft	Regulation	regulation of biological quality (GO:0065008)	702	9
		regulation of gene expression (GO:0010468)	2409	7
	Cytoskeletal	tubulin binding (GO:0015631)	189	4
	Organelle	cilium assembly (GO:0060271)	146	4
	Signaling	cytokine receptor activity (GO:0004896)	74	3
	Metabolism	oxidoreductase activity (GO:0016616)	88	3
		fatty acid beta-oxidation (GO:0006635)	28	3
		protein folding (GO:0006457)	102	3
Up regulated in hard	Autophagy	autophagy of mitochondrion (GO:0000422)	34	3
		autophagosome assembly (GO:0000045)	46	3
		phagophore assembly site (GO:0000407)	25	3
	Cell-cell Interaction	cell migration (GO:0016477)	213	7
		extracellular matrix organization (GO:0030198)	122	5
		focal adhesion (GO:0005925)	52	4
		extracellular matrix structural constituent (GO:0005201)	50	3
	Cytoskeletal	actin filament binding (GO:0051015)	157	5
	Metabolism	ligase activity (GO:0016874)	138	5
		mannosyltransferase activity (GO:0000030)	15	3
		cysteine-type endopeptidase activity (GO:0004197)	44	3
		protein N-linked glycosylation (GO:0006487)	37	3
	Regulation	GTPase activator activity (GO:0005096)	156	6
		positive regulation of transcription (GO:0045944)	230	6
		intracellular monoatomic cation homeostasis (GO:0030003)	137	5
		positive regulation of kinase activity (GO:0033674)	73	4
		positive regulation of peptidase activity (GO:0010952)	28	3
		catalytic step 2 spliceosome (GO:0071013)	54	3
	Signaling	transmembrane signaling receptor activity (GO:0004888)	1044	5
		BMP signaling pathway (GO:0030509)	23	3
	Transport	P-type ion transporter activity (GO:0015662)	18	3

## Discussion

NFPAs are benign tumors of the CNS that usually affect patients in their fifth or sixth decades of life and can cause significant symptoms such as severe headaches, visual deficits (i.e., bitemporal hemianopsia or oculomotor weakness), and even hypopituitarism from compression of the normal gland, all of which can be debilitating to the daily activities of this patient population [16]. Endoscopic endonasal resection provides a corridor for diagnosis and potential for cure [14,16].

Despite being generally considered a safe, minimally invasive procedure with very high cure rates, surgical resection is still an invasive treatment modality with the potential for neurovascular injury that can lead to hypopituitarism, blindness, ophthalmoplegia, and even stroke [14,17]. The effects of hypopituitarism from a patient's perspective cannot be underscored enough. In fact, in a recent retrospective postoperative survey of patients, the majority surveyed preferred to have tumor recurrence with sparing of the normal gland rather than total tumor resection with the need for hormonal therapy, a potential consequence of more aggressive surgical resections [18]. With the growth of new targeted gene therapies and immunotherapies for certain more invasive and malignant tumors of the CNS, other non-surgical treatments for residual and recurrent NFPAs may potentially have a future role [1,3].

With 60-70% of pituitary adenomas being non-functional, understanding NFPA intratumoral genomic heterogeneity may provide significant insight into tumor behavior that could prognosticate future recurrence and invasiveness [19]. For example, Yuan et al. demonstrated great evidence of a potentially targetable gene (ANXA-2) and the usefulness of gene expression modeling in exploring the basis of pathogenesis in NFPAs (e.g., the h-PASC gene) [13]. Similarly, there is likely substantial merit in understanding the intratumoral heterogeneity of gene expression seen in the more phenotypically visible intraoperative features, such as texture, firmness, adhesiveness, and growth location.

Intratumoral genetic heterogeneity can help elucidate the origins of the tumor’s biological pathogenesis and phenotypic behavior. A 2017 genomic study of meningiomas by Dewan et al. suggested significant intratumoral genetic heterogeneity (in mutations of ADAMTSL3 and CAPN5) in grade II meningiomas that can affect tumor hardness, fibrosity, and adhesiveness [20]. These phenotypic changes can necessitate different surgical resection techniques. These findings highlight the importance of tumor sample localization and texture. Furthermore, the determination of differential gene expressions within the tumor based on tumor marginality (edge vs. core) and texture (firm vs. soft) may indicate an important role in the holistic understanding of NFPA pathogenesis. Perhaps these findings may also yield explanations for clinically noted differences in adenoma invasiveness, adhesiveness, and sites of tumor recurrence [21]. 

In this single-center study, we reviewed the transcriptomic heterogeneity of gene expression across eight surgical resection samples from four patients (two samples each, one from the core and one from the tumor margin). Studied in both the radiological and surgical literature, NFPAs generally consist of a peripherally firmer capsule that encompasses a softer and more necrotic core [22]. As dedicated pituitary preoperative imaging of our patients shows areas of different tumor intensity within the tumor, we hypothesized that there are likely differential transcriptomic gene expressions based on the anatomic marginality (core vs. edge) of the NFPA and within the different texture phenotypes. 

Transcriptomic findings showed 67 genes that were differentially expressed across the anatomic marginality of tumor samples (edge vs. core). The ontologic classification of these genes recognized gene functionalities within cell signaling, cell cytoskeleton, and cell division, features that may correlate with the biologically less invasive, slower-growing, yet firmer portion of the NFPA’s edge or capsule. Specifically, genes involved in cell-cell adhesion, such as cadherin-binding proteins, were enriched 10-fold in the edge samples. Although there were also genes with transport functionality that were differentially expressed, most of these genes were mutually exclusive with the cell cytoskeleton genes. Future studies are needed to study the proteomic interactions of the individual differentially expressed genes on a case-by-case basis. Although a very preliminary view, identifying and targeting unique cadherin-binding proteins of the tumor edge can potentially reduce the fibrotic nature of this portion of the tumor, allowing for easy suctioning and removal intraoperatively.

The transcriptomic analysis of samples based on their intraoperative tumor texture findings (firm vs. soft) showed differential expression in gene functionalities such as cell-cell interaction, autophagy, and cell cycle regulation. However, the cell-cell interaction genes were not as significantly different as compared to the differences found in the tumor marginality samples. Uniquely differential upregulation of cell division genes (such as cilium assembly, tubulin binding, and cytokine receptor activity) and aerobic metabolism genes (such as fatty-acid beta-oxidation and oxidoreductase) in hemorrhagic, well-oxygenated, firm tumors compared to their softer counterparts can potentially be associated with the biology of rapidly expansive necrotic tissue in the softer tumors. Indeed, this was also evident in the differential expression of autophagy genes (such as phagophore and autophagosome assembly) and extracellular matrix genes (such as actin filament binding and other extracellular constituents) in the firm tumors, which may imply the slower-growing but more organized, well-pruned, well-adhered, and more stable tumor cell colonies. Similar to the tumor edge, identification of unique autophagy cascade and aerobic metabolism genes and proteins in the firm tumor may allow for the discovery of niche targetable vulnerabilities, allowing for transition out of aerobic metabolism and autophagy to soften the tumor prior to resection. Certain drugs, such as hydroxychloroquine, have been previously used to target autophagy in the past, but more targeted immunotherapies are still under continued investigation. The authors would like to caution readers on the preliminary nature of these suggestions only as potential for future studies.

As a form of general control for interpatient heterogeneity and involvement of variations between the origin of the NFPA cells across patients, we also performed an interpatient transcriptomic analysis. This portion of the study yielded 164 differentially expressed genes that were mostly (~80%) grouped into metabolic function genes by ontologic categorization. The remainder of these genes were mostly part of the brain-specific neurotransmitter recycling (such as amyloid beta-binding protein and neurotransmitter-binding proteins). Although there have been previous whole-body genomic speculations about the involvement of metabolic risk factors in patients with functioning pituitary adenomas, these hypotheses have not been fully developed in NFPAs and have never been tested experimentally [23]. However, the specific findings of differential transcriptomic expression in certain metabolic genes (such as pyrophosphate, nucleoside triphosphatase, and hydrolase activities) within the context of our study across patients with similar backgrounds and pathologies of NFPAs are evidence of significant variations in the biology of cell fuel production pathways and metabolism. Although this finding is currently of no clinical significance, future, more focused studies can potentially address the metabolic gene networks of NFPAs for targeted gene therapies.

Limitations of this study included being a non-randomized, non-controlled, single-centered study of limited sample size (only eight samples from four patients), therefore limiting its demographic generalizability. Given our limited population size, it was also not possible to control for age, gender, or other demographic factors. Despite comparing our results from only eight samples across different tumor features (marginality and texture), we did find statistically and clinically novel and significant findings about the intratumoral genetic heterogeneity of NFPAs. This study takes the novel approach of using high-resolution transcriptomics analysis of intramural genomic expression heterogeneity with statistically significant findings. We decided to keep our ontologic explanations and findings broad to avoid tendency explanation bias and an over-granular breakdown of every gene’s enrichment without enough clinical power. In the future, we would like to expand this study to include more patients and samples to enhance the generalizability of our findings.

In an overview of the ontologic findings of this study, we noted that a majority of the differential genes belonged to the metabolism category, followed by cell division, cell transport, cytoskeleton, and cell-cell interaction categories. The majority of the differentially expressed metabolism genes belonged to the interpatient analysis, which needs higher power studies to appreciate the granular contribution of excitatory and inhibitory patterns of genes to pinpoint possible areas of microbiologic intervention and targeted therapy. We hope that the findings of this study can provide grounds for more extensive investigations through granular gene regulatory network modeling of many other clinically important NFPA features such as tumor invasiveness, adherence, and recurrence, and identify commonalities of differential gene expression for targeted gene therapies.

## Conclusions

This single-center prospective transcriptomic study of eight NFPA samples from four patients showed significant yet unique gene expression and intratumoral genetic heterogeneity across tumor texture, location, and patients. Soft tumors were more likely to upregulate their anaerobic metabolism and cell division genes, whereas firm tumors were more likely to uphold their organized and dense cell structures through the upregulation of autophagy and extracellular matrix genes. The anterior sellar surface of the NFPA had a 10 times enrichment of cadherin-binding proteins, cell adhesion proteins, and intracellular cytoskeleton proteins compared to that found within the core of the tumor. These findings were different from the interpatient differential gene expression of NFPAs, which most significantly involved genes of cell metabolism. Future studies on the gene regulatory networks that influence NFPA consistency, invasiveness, and growth may provide individualized targets for disruption.
